# Correction: A comprehensive review about immune responses and exhaustion during coronavirus disease (COVID-19)

**DOI:** 10.1186/s12964-022-00967-4

**Published:** 2022-09-08

**Authors:** Rebar N. Mohammed, Rozita Tamjidifar, Heshu Sulaiman Rahman, Ali Adili, Shadi Ghoreishizadeh, Hossein Saeedi, Lakshmi Thangavelu, Navid Shomali, Ramin Aslaminabad, Faroogh Marof, Mina Tahavvori, Svetlana Danshina, Morteza Akbari, Gülinnaz Ercan

**Affiliations:** 1 Medical Laboratory Analysis Department, College of Health Sciences, Cihan University of Sulaimaniya, Kurdistan Region, Iraq; 2 College of Veterinary Medicine, University of Sulaimani, Sulaimaniyah, Iraq; 3 I.M. Sechenov First Moscow State Medical University, Moscow, Russia; 4 Department of Physiology, College of Medicine, University of Sulaimani, Sulaimaniyah, Iraq; 5 Department of Medical Laboratory Sciences, Komar University of Science and Technology, Sarchinar District, Sulaimaniyah, Iraq; 6 Department of Oncology, Tabriz University of Medical Sciences, Tabriz, Iran; 7 Immunology Research Center, Tabriz University of Medical Sciences, Tabriz, Iran; 8 Department of Pharmacology, Saveetha Dental College, Saveetha Institute of Medical and Technical Science, Saveetha University, Chennai, India; 9 Department of Medical Biochemistry, Faculty of Medicine, Ege University, 35100 Izmir, Turkey; 10 Department of Stem Cell, Institute of Health Sciences, Ege University, Izmir, Turkey

## Correction: Cell Communication and Signaling 20(1):79 (2022) https://doi.org/10.1186/s12964-022-00856-w

Following publication of the original article [1], the authors identified an error in the author name of Svetlana Danshina.

The incorrect author name is: Svetlana Danishna

The correct author name is: Svetlana Danshina

The author group has been updated above and the original article [1] has been corrected.
